# Beyond the Biomarker: Monomeric CRP as a Driver of Multisystem Pathology in Rheumatoid Arthritis

**DOI:** 10.3390/ijms26178227

**Published:** 2025-08-25

**Authors:** Andreea Lazarut-Nistor, Mark Slevin

**Affiliations:** 1 Rheumatology Clinic, County Emergency Clinical Hospital, 540136 Targu Mures, Romania; andreealazarut@yahoo.com; 2 Centre for Advanced Medical and Pharmaceutical Research, George Emil Palade University of Medicine, Pharmacy, Science, and Technology of Targu Mures, 540142 Targu Mures, Romania

**Keywords:** rheumatoid arthritis, monomeric CRP, cardiovascular disease, neurodegenerative disease, inflammation

## Abstract

Chronic inflammation underpins the pathogenesis of both rheumatoid arthritis (RA) and neurodegenerative conditions such as Alzheimer’s disease (AD). This narrative review explores the role of C-reactive protein (CRP), particularly its monomeric form (mCRP), as a central molecular link connecting systemic autoimmune inflammation with neuroinflammatory and vascular pathology. In RA, fibroblast-like synoviocytes (FLSs) are activated by CRP through CD32/CD64-mediated signaling, triggering proinflammatory cascades involving NF-κB and p38 MAPK. Recent studies have highlighted that locally synthesized CRP within the synovium may convert to mCRP, amplifying inflammation and tissue damage. Beyond RA, mCRP has been identified within amyloid-beta (Aβ) plaques in AD brains, suggesting a direct role in neurodegenerative pathology. Experimental models also demonstrate that mCRP is upregulated in stroke-affected brain regions and associated with complement activation and blood–brain barrier (BBB) disruption, which is central to AD progression. The convergence of pathways involving IL-6, RAGE (receptor for advanced glycation end-products), and mCRP-mediated complement activation reveals a shared axis of inflammation between RA and AD. This highlights the potential of mCRP not only as a biomarker of chronic inflammation but also as a therapeutic target. Furthermore, evidence from periodontal disease and cardiovascular comorbidities highlights the systemic nature of mCRP-driven inflammation, offering insights into the mechanisms of disease overlap. This review advocates for further mechanistic studies into mCRP signaling, particularly its role at the interface of systemic and neuroinflammation, with the goal of identifying new interventional strategies for patients with RA at elevated risk of neurodegenerative and vascular complications.

## 1. Introduction

Rheumatoid arthritis (RA) is an autoimmune condition characterized by progressive destruction of the joints. The main site of inflammation in RA is the joint, and, here, the primary architecture, matrix and micro-environment become obliterated by inflammation of the synovial membrane. A critical biomarker used in monitoring disease progression and response to treatment is C-reactive protein (CRP). CRP is an acute phase reactant which exists in at least three conformational forms: the native circulating soluble pentameric CRP (pCRP) form, pentameric symmetrical form (pCRP*) and the tissue insoluble plasma bound monomeric CRP (mCRP) [1]. In clinical practice, CRP is commonly measured in its native, serum-soluble form (pCRP) and is included in tools like the Disease Activity Score 28 (DAS28-CRP) to guide treatment decisions. However, normal CRP levels do not always reflect the absence of disease activity, leading to potential delays in treatment during flares [2].

Kim et al. examined how CRP (native versus monomeric not distinguished in the tissue) was able to predict RA progression and inflammation in synovial tissue obtained from seven RA and five osteoarthritis (OA) patients and assessed the expression of CRP in RA synovium through immunohistochemical staining. Serum pCRP levels were correlated with synovial fluid (SF) CRP levels, revealing that SF-CRP was higher in the RA group compared to OA patients. CRP was expressed in significant quantities within the lining and sub-lining of the synovium, and whilst its origin was presumably associated with excessive chronic production and systemic release from the liver hepatocytes, the capacity of CRP (and primarily mCRP) to ‘piggy back’ onto other cells within the blood such as monocytes and lymphocytes could represent a mechanism through which it targets the inflamed tissue. Mechanistically, the authors found that the receptor activator of nuclear factor kappa-light-chain-enhancer of activated B cells (NF-κB) ligand (RANKL), produced by monocytes, induced osteoclastogenesis (the process of formation and differentiation of osteoclasts from precursor cells, inducing bone remodeling) in RA. Systemic inflammation (characterized by raised serum pCRP levels) increases the levels of pro-inflammatory cytokines, which, in turn, stimulate RANKL expression on osteoblasts and immune cells, promoting osteoclastogenesis and bone resorption. When CD14+ monocytes were isolated from peripheral blood and stimulated with pCRP, both RANKL production and expression increased. These findings suggest that reducing CRP levels in RA could help prevent bone destruction [3].

The observation that CRP accumulates in RA synovial tissue and actively contributes to inflammation and osteoclastogenesis confirms its role as more than a passive biomarker; it is a functional participant in the inflammatory cascade. Given that CRP, particularly in its monomeric form, not only exacerbates local joint pathology but also circulates systemically in elevated concentrations, its involvement in extra-articular manifestations becomes increasingly relevant. Among these, one of the most clinically significant is cardiovascular disease (CVD), which disproportionately affects patients with RA. This elevated risk is not merely coincidental but stems from shared inflammatory pathways, including those involving CRP, RANKL, and the receptor for advanced glycation end products (RAGE), linking persistent systemic inflammation with vascular dysfunction and atherosclerosis.

RA significantly increases cardiovascular (CV) risk due to systemic inflammation, immune dysregulation, and associated molecular pathways. A key contributor is RAGE, which, when activated by proinflammatory ligands such as S100A8/A9 and S100A12, intensifies the body’s inflammatory signaling and cellular responses in both joints and blood vessels [4]. Alterations in HDL composition and function, especially reductions in small, atheroprotective HDL particles, link lipid metabolism to inflammation [5], whilst elevated CRP and other markers like osteopontin (OPN) correlate with arterial stiffness, vascular dysfunction, and CV events. These overlapping pathways and interactions correlating with disease progression will be described in more detail later in this review [6]. Functional vascular changes often precede structural abnormalities and can be detected through imaging and biomarkers, including reduced endothelial repair capacity via decreased expression of CD34+ progenitor cells, providing at least an indication of antagonism or distress; however, a complete understanding of the physiological interactions associated with inflammation-driven vascular damage in RA is required (and still lacking) in order to interpret and provide early and targeted interventions to reduce CV morbidity and mortality [7]. CRP/mCRP has also been increasingly implicated in the pathogenesis of Alzheimer’s disease (AD) through its association with development of beta-amyloid (Aβ) plaques and neuroinflammation [8]. Emerging evidence also links systemic inflammation, vascular dysfunction, and specifically, autoimmune conditions like RA and periodontal disease to cognitive decline and depression, suggesting a broader role of chronic inflammation in neurodegenerative processes [9].

Therefore, we reviewed the existing literature on the role of CRP and more specifically mCRP in RA with the purpose of analyzing evidence supporting a potential its role in prognosis and treatment of RA patients with joint inflammatory disease, and a novel potential role in predicting RA-associated cardiovascular and neurocognitive complications.

## 2. CRP and mCRP in RA

### 2.1. Physiology of CRP and mCRP Conversion in Vascular Disease

The reason CRP levels are important to register in autoimmune diseases such as RA was underscored by a study which investigated whether sustained suppression of inflammation, as measured by CRP levels, more effectively prevents the development of new joint damage than the progression of existing damage in RA. Over a 5-year period, 359 patients with active RA were monitored, with CRP levels and radiographic joint damage assessed regularly. The results showed a strong correlation between higher time-averaged CRP levels and increased joint damage, particularly in patients with early RA. Notably, new joint involvement rose sharply with higher CRP (a 5.4-fold increase), whereas progression in already damaged joints increased to a lesser extent (1.6-fold). These findings suggest that early and sustained control of inflammation is critical to preventing the spread of joint damage in RA [10]. In another study, radiographic progression after one year was associated with severe initial joint damage, elevated CRP levels, and the presence of IgM rheumatoid factor at baseline [11]. To further support this, in a group of 109 patients with normalized CRP, functional improvement persisted unless CRP levels rose again, concluding that CRP suppression is associated with improved function and that elevated CRP can serve as a useful short-term predictor of functional outcomes and a therapeutic guide in early RA [12].

CRP undergoes conformational changes upon encountering injured tissue. Native CRP, which is pentameric in structure, dissociates into its monomeric subunit form (mCRP) at sites of inflammation. Interestingly, an intermediate conformation known as pCRP*, which exposes a neoepitope, can also be found in inflamed tissues. This conformational change plays a critical role in immune activation. In a study by Fujita et al. [1], plasma mCRP was shown to be a more specific and sensitive biomarker than pCRP for diagnosing the rare autoinflammatory type of arthritis Adult-Onset Still’s Disease (AOSD), with significantly elevated levels in affected patients. Braig et al. [13] revealed that native CRP can bind to activated monocytes and microvesicles, particularly on disturbed phospholipid membranes. This study showed that pCRP was bound specifically to LPS-activated THP-1 cells and human monocytes, as confirmed by flow cytometry (BD LSR Fortessa Cell Analyzer, BD Bioscience, San Jose, CA, USA) and confocal microscopy (Leica TCS SP2 AOBS; Leica Microsystems, Wetzlar, Germany) and processed with the Leica Confocal Software. pCRP was seen to form clusters on the plasma membrane and subsequently complexes with microvesicles (MVs). Mechanistically, Western blots revealed NF-κB pathway activation and mCRP in supernatants, indicating pentamer dissociation. Pentameric CRP binding was time- and sequence-dependent. These findings suggest a regulated role for pCRP-mCRP dissociation status in coordinating inflammatory responses. Conformational analyses revealed that CRP on MVs can adopt both pCRP and mCRP forms, unlike intact cells where only pCRP is detected. This binding pattern was confirmed across MVs from various immune cells and observed in circulating MVs from patients with ST-elevation myocardial infarction, highlighting the physiological relevance of pCRP-MV interactions [13].

CRP exists in two main isoforms: pentameric CRP and monomeric CRP, which differ markedly in their structure, localization, biological activity, and detection. pCRP is the native, circulating form composed of five identical subunits, primarily synthesized by the liver in response to IL-6 [14,15]. It serves as a widely used systemic marker of inflammation, activating the classical complement pathway and binding to phosphocholine on microbial surfaces and apoptotic cells. In contrast, mCRP is generated at sites of inflammation through the dissociation of pCRP, often triggered by oxidative stress, activated cell membranes, or bioactive lipids [16]. This monomeric form is structurally and functionally distinct, exerting potent pro-inflammatory effects by promoting endothelial dysfunction, inducing cytokine and adhesion molecule expression, and enhancing leukocyte and platelet activation. While pCRP is readily measured using standard immunoassays, the detection of mCRP poses significant challenges due to its conformational differences and the lack of standardized assays, often leading to its underrepresentation in clinical studies. Importantly, mCRP is increasingly recognized not just as a byproduct but as an active mediator of local inflammation in various pathological conditions, including atherosclerosis, neurodegenerative diseases, and autoimmune disorders, underscoring the need to distinguish between CRP isoforms in both research and clinical settings [17,18].

Extracellular vesicles (EVs) play a key role in several pathogenic mechanisms underlying the development of RA. These include promoting immune complex formation, facilitating antigen presentation, transporting microRNAs, inflammatory cytokines, proteases, and other proteins, activating fibroblast-like synoviocytes (FLS), enabling intercellular communication, and contributing to extracellular matrix degradation. Notably, differences in the content of EVs derived from synovial fluid and plasma may serve as valuable disease biomarkers. Elevated levels of EVs have been observed in the plasma of individuals with RA, and the presence of citrullinated proteins on the EV membrane has emerged as a potential RA-specific biomarker. The build-up of immune complexes in tissues is believed to be a central factor contributing to inflammation in autoimmune diseases [19]. Further research has revealed that EVs present in synovial fluid, derived from multiple cell types, can form immune complexes. Notably, a large proportion of these EV-associated immune complexes express CD41, a platelet marker, suggesting that many of these immunogenic EVs originate from platelets [20].

A review by Schioppo et al. examined 41 studies focusing on EVs, the membrane-bound vesicles involved in cell-to-cell communication known to be impaired in RA. Among the studies, 4, involving a total of 180 patients, found that the number of plasmatic EVs was higher in individuals with RA compared to controls, while 3 other studies, covering 74 RA patients, reported similar EV levels between groups. Overall, the conclusion drawn implied that the total concentration of plasmatic EVs is increased in RA patients compared to controls, as well as those with osteoarthritis and reactive arthritis [21]. The identification of platelet-derived EVs (expressing CD41) forming immune complexes in RA synovial fluid suggests an active contribution to immune-complex-mediated inflammation, a process in which mCRP is known to enhance opsonization and complement fixation. These observations collectively support the hypothesis that CRP/mCRP may not only reflect systemic inflammation in RA but may actively participate in EV-driven local immune activation, indicating a potential role as effectors of disease progression.

Since the neoepitope is exposed in mCRP, anti-mCRP antibodies have been used to identify tissue-deposited CRP. Notably, these structural transitions enable the activation of the complement system; for instance, C1q complement factor binds to pCRP* but not to intact pCRP. The accessibility of the C1q binding site is critical for complement activation, which is only possible once CRP transitions into the monomeric form. In RA, complement activation occurs due to upregulated pro-inflammatory signaling, which subsequently increases CRP synthesis. Therefore, the high levels of CRP, depending on its conformation, most likely contribute to disease progression through both cellular signaling and complement modulation [22].

### 2.2. The Dual Role of CRP-mCRP in Pathological Development of RA

Rheumatoid arthritis is a systemic autoimmune disease that primarily targets synovial joints and may be initiated by environmental triggers. Synoviocytes, which respond dynamically to circulating cytokines, play a central role in translating external inflammatory signals into local cellular responses within the joint environment. Among these cytokines, tumor necrosis factor-alpha (TNF-α), primarily secreted by activated macrophages, plays a central pathogenic role. This was demonstrated by Jia et al., who showed that transgenic mice overexpressing TNF-α spontaneously developed erosive arthritis, closely mimicking human RA pathology. To investigate the role of CRP in bone erosion, the study examined the effects of nCRP and mCRP on osteoclast differentiation and bone resorption. Using murine macrophage cell lines and bone-marrow-derived macrophages, endotoxin-free CRP proteins and peptides were applied either alone or in combination with RANKL. Through this approach, they found that nCRP had no effect on osteoclast differentiation, while mCRP became intracellularly localized, significantly promoting osteoclastogenesis across multiple cell types. Since the number of osteoclasts increased after mCRP was given by subcutaneous injection on calvaria to healthy mice, the study showed that osteoclast differentiation occurs when CRP is in the monomeric conformation. Enzyme-linked immunosorbent assay (ELISA) demonstrated that mCRP bound directly to RANKL, suggesting a possible mechanism for its osteoclast-promoting activity. In vivo, inflammatory osteolysis was induced in wild-type and CRP knockout mice, and histological analysis revealed that bone destruction was more pronounced in the presence of mCRP. These findings collectively indicate that CRP influences osteoclast differentiation in a conformation-dependent manner, with mCRP (unlike the native molecule) playing a role in bone resorption in inflammatory conditions such as RA, implying that mCRP–RANKL interactions could be explored as therapeutic targets for osteoclast modulation in RA [23].

On the other hand, several studies have questioned the pathogenic role of mCRP and its significance compared to pCRP. Experimental work has shown that many pro-inflammatory effects attributed to mCRP may be artefactual, resulting from contamination with endotoxin or sodium azide in CRP preparations [24]. For instance, a study comparing native and denatured CRP found no pro-inflammatory activity from mCRP in endothelial cells, contradicting earlier claims and attributing previous findings to impurities [25]. Similarly, reviews have emphasized that the conditions used to generate mCRP in vitro often do not reflect physiological processes, and many findings may lack in vivo relevance [26]. Animal studies using CRP-transgenic models have yielded conflicting results, with several showing no proatherogenic effect, and in some cases, mCRP even exerting anti-inflammatory activity via upregulation of IL-10. Furthermore, large-scale Mendelian randomization studies have demonstrated that genetically elevated CRP levels do not associate with increased cardiovascular disease risk, undermining the case for CRP (in either form) as a causal factor [27]. Collectively, this body of work suggests that mCRP may not be as pathogenic as previously thought, or may be more ‘selectively’ pathogenic, and hence its biological significance relative to pCRP remains unresolved to date.

Detecting mCRP in vivo remains challenging due to several key limitations, particularly regarding antibody specificity and isoform stability. Major obstacles include the lack of antibodies that can reliably distinguish mCRP from pCRP and the absence of standardized, clinically validated assays for mCRP detection. Many commercially available antibodies exhibit cross-reactivity, recognizing shared epitopes between the isoforms, especially under conditions where pCRP partially dissociates or denatures [16,28]. This lack of specificity can lead to inaccurate localization or quantification of mCRP in tissues. Additionally, mCRP is structurally unstable under physiological conditions; unlike the stable, circulating pCRP, mCRP is typically generated at sites of inflammation and rapidly associates with membranes or extracellular components, making it poorly soluble and difficult to detect in plasma or serum [29]. Moreover, since mCRP is primarily localized at inflammatory sites rather than circulating freely in blood, conventional biofluid-based assays may not reflect its true pathological burden, necessitating invasive tissue sampling or advanced imaging for accurate assessment [30].

Another important inflammatory signaling protein is interleukin-6 (IL-6), which plays a central role in RA by driving chronic joint inflammation, promoting synovial hyperplasia and pannus proliferation via endothelial growth factor (VEGF), stimulating B and T cell activation leading to autoantibody production, enhancing osteoclast-mediated bone erosion, contributing to systemic manifestations such as fatigue, anemia, and elevated acute-phase proteins (by promoting CRP production in hepatocytes). When neutrophils migrate from blood to the synovium, they release proteolytic enzymes and reactive oxygen intermediates, leading to joint inflammation, further attracting leukocytes and contributing to monocyte infiltration [31]. Because IL-6 is central to both systemic and joint specific inflammation, IL-6 inhibitors can effectively reduce CRP levels and systemic inflammation.

C-reactive protein plays a dual role in RA pathogenesis. During the early and pre-clinical stages of RA, as well as in models of experimentally induced arthritis, CRP may exert beneficial effects, possibly through its role in immune complex formation and complement activation. However, in established, active RA, CRP contributes to joint damage and inflammation. Importantly, a study by Jones et al. revealed that in collagen-induced arthritis (CIA) mouse models, which resemble human RA, CRP appeared beneficial without affecting the autoantibody profile. This challenges the conventional view linking high CRP levels with worsening RA symptoms, proposing instead that elevated CRP during active RA reflects a response aimed at dampening inflammation. The study highlights the need to reconsider CRP’s role in arthritis, emphasizing its potential protective role before disease onset and encouraging further research to develop CRP based therapeutic strategies. This study compares wild-type, CRP-deficient, and CRP-transgenic mice to investigate the role of CRP in inflammation and immunity, particularly in CIA. The findings show that CRP is not merely associated with inflammation but actively regulates immune responses at multiple levels, including cytokine production, hypersensitivity, and antibody responses. CRP deficiency leads to a more rapid and severe progression of CIA, while CRP overexpression slows disease progression and reduces symptoms. The protective effects of CRP likely involve accessory immune cells and the FcγRIIB receptor pathway. These mouse model results suggest CRP has a tonic, baseline suppressive effect on inflammation that helps prevent autoimmunity [32]. However, this finding does not refer to mCRP but to nCRP and needs to be further supported.

The monomeric form of CRP is primarily responsible for pro-inflammatory activity, while the pentameric form of CRP generally exhibits anti-inflammatory properties. The transition from pCRP to mCRP is triggered by binding to damaged membranes or inflammatory microenvironments. This process produces an intermediate (pCRP*) that activates the classical complement cascade and ultimately converts to mCRP, which enhances immune responses such as platelet aggregation, neutrophil migration, cytokine release, and NK cell activation. Conversely, pCRP can inhibit platelet and neutrophil activity, reflecting its regulatory role. CRP isoforms interact with different Fc receptors, contributing to their opposing effects. The transition from pCRP to mCRP is fast and localized, but slows to prevent systemic inflammation, with detectable pCRP levels rising in serum 6–12 h after tissue injury [33]. CRP’s conformational change from pCRP to mCRP enhances leukocyte recruitment and reactive oxygen species generation in ischemia/reperfusion injury. This transition occurs when pCRP dissociates into monomers, typically in response to tissue damage or other inflammatory stimuli. The structural shift is significant as mCRP exhibits pro-inflammatory properties, such as platelet activation, leukocyte recruitment, and endothelial dysfunction, which contribute to the pathogenesis of various diseases, including RA and cardiovascular conditions [34].

The complement (C) system is a critical component of the innate immune response, consisting of approximately 60 proteins found in serum and on cell membranes. It functions alongside antibody-mediated reactions to defend against microbial infections and helps clear damaged tissues and cellular debris. The system operates through three activation pathways: the classical (antibody-triggered), lectin (recognizing microbial carbohydrate patterns), and alternative (continuously active, antibody-independent) pathways, all converging on the activation of C3 and deposition of C3b. Tight regulation of complement activity is essential, as dysregulation can contribute to various diseases including infections, cancer, renal disorders, and autoimmune conditions. In RA, evidence suggests that inappropriate activation of the complement system plays a role in disease pathogenesis. Given the complement system’s involvement in inflammation and tissue damage, it represents a promising area for biomarker discovery and therapy development. A study by Rodríguez-González et al. comprehensively evaluated all three complement (C) pathways: classical, alternative, and lectin in 430 patients with RA, using both functional assays and measurements of individual complement proteins. The findings showed that RA disease activity was primarily associated with upregulation of the terminal pathway, particularly through the classical cascade. Higher disease activity of RA correlated with increased functional test values, indicating reduced consumption and increased hepatic production of complement components. CRP demonstrated strong correlations with the classical and terminal pathways but weaker associations with the lectin pathway, supporting its role in activating the classical cascade via C1q. Lectin pathway deficiency, a common condition affecting 5–30% of the general population, has been linked to more severe or erosive RA. The presence of RA and ACPA was associated with lower levels of classical pathway components, possibly reflecting local complement consumption in inflamed joints. Although complete inhibition of the C system has not shown efficacy in RA, the findings in this study suggest that targeted or partial complement modulation may still offer therapeutic benefit [35].

In addition, complement activation appears to be localized during the preclinical phase and becomes systemic with the onset of clinical RA, suggesting it contributes to disease progression rather than initiation [36]. Confirming this, one study which examined 107 patients with active RA and 177 patients with inactive RA displayed strong evidence that CRP contributes directly to complement activation in RA, as demonstrated by elevated levels of CRP–complement complexes (C3d-CRP and C4d-CRP), particularly in patients with active disease. The significant correlation between these complexes and disease activity scores (DAS28) supports the idea that CRP-mediated complement activation is not only present but also linked to disease severity. This suggests that CRP is more than just a marker of inflammation in RA and that it may actively participate in the disease’s pathogenesis through its role in complement activation [37]. Another study identified CRP as a potential contributor to complement activation on the surface of microparticles, particularly in plasma. Microparticles isolated from the plasma of both RA patients and healthy individuals showed a strong correlation between CRP binding and C1q binding, implicating CRP in the initiation of the classical complement pathway. CRP is known to bind to phosphorylcholine and oxidized phosphatidylcholine on cell membranes, especially in the presence of lysophosphatidylcholine, which may be present on microparticles due to oxidative stress and increased enzymatic activity. Interestingly, although fluid-phase CRP levels were elevated in both plasma and synovial fluid of RA patients, complement activation was associated specifically with microparticle-bound CRP, suggesting that its role in complement activation is localized to surfaces rather than driven by soluble CRP levels. These findings underscore CRP’s potential function as a surface-bound activator of complement on microparticles and highlight its possible contribution to systemic inflammation in RA [38].

CRP plays a key role in innate immunity by binding to phosphocholine (PC) on apoptotic cells and pathogens and interacting with complement factor C1q and Fcγ receptors. In its native pentameric form, CRP is not inherently pro-inflammatory. However, as stated previously, under certain conditions (e.g., heat, acidic environments, or phospholipase A2 activity), pCRP can dissociate into mCRP or an intermediate form (pCRP*), both of which expose neoepitopes and exhibit strong pro-inflammatory properties. Dissociation alters the structure and solubility of CRP, making mCRP largely tissue-bound. Microparticles from activated cells can transport dissociated CRP forms to distant sites, including injured or inflamed tissues. While pCRP is mostly inactive, both mCRP and pCRP* are potent activators of inflammation and thrombosis. Plasma CRP levels, which can rise dramatically during inflammation, serve as a widely used clinical marker of systemic inflammation [39] and are used to monitor disease activity in RA patients.

### 2.3. CRP Signaling in RA Synovial Inflammation

**Figure 1:**
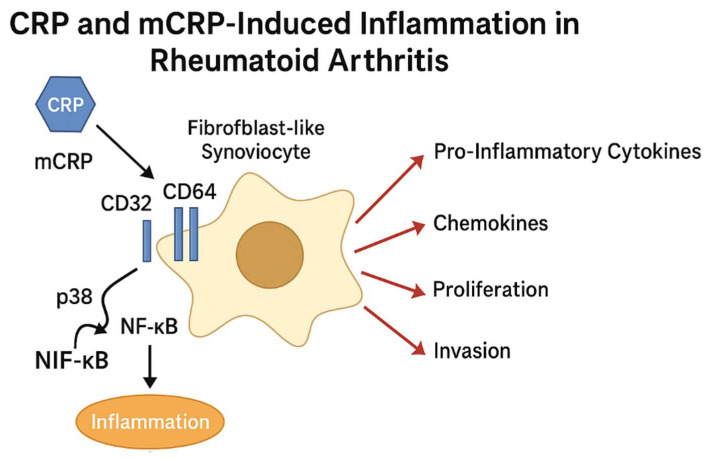
Schematic representation of CRP and mCRP-induced inflammatory signaling in fibroblast-like synoviocytes in rheumatoid arthritis. This diagram illustrates the proposed mechanisms by which CRP and its monomeric form (mCRP) contribute to synovial inflammation in RA. In the RA synovium, FLSs express elevated levels of CRP receptors, including CD32 (Fc gamma receptor II) and CD64 (Fc gamma receptor I), both of which are minimally expressed in healthy controls. Locally produced CRP and mCRP interact with these receptors on FLSs, triggering intracellular signaling cascades. Specifically, CRP binding to CD32 and/or CD64 leads to the activation of the p38 mitogen-activated protein kinase (p38 MAPK) pathway and the NF-κB pathway. Activation of these signaling intermediates results in the phosphorylation of downstream targets and the transcription of pro-inflammatory genes. This cascade promotes the secretion of pro-inflammatory cytokines and chemokines and enhances the proliferative and invasive phenotype of RA-FLSs. These cellular responses contribute to the amplification of synovial inflammation and joint destruction characteristic of RA. Pharmacologic inhibition of NF-κB, for example, with pyrrolidine dithiocarbamate (PDTC), has been shown to suppress CRP-induced FLS proliferation, further supporting the relevance of this pathway. The figure highlights the pathogenic role of CRP and mCRP in mediating synovial inflammation through receptor-mediated activation of p38 MAPK and NF-κB in RA.

A study by Fang et al. analyzed synovial tissue from 21 patients with RA and 3 healthy controls to investigate the role of CRP in synovial inflammation. Synovial tissues from RA patients exhibited markedly elevated CRP signaling compared to controls. Notably, fibroblast-like synoviocytes (FLSs) were identified as a major cell type responsive to CRP stimulation. RA-FLSs demonstrated increased proliferative and invasive capacities in response to CRP exposure, alongside elevated expression of pro-inflammatory cytokines and chemokines. These cells also exhibited morphological alterations and expressed several inflammation-related markers, including major histocompatibility complex class II (MHC-II), adhesion molecules, proangiogenic factors, and matrix-degrading enzymes. To determine whether CRP is locally produced in the RA synovium, two-color immunofluorescence was used to assess CRP expression. CRP was found to be highly expressed in RA synovial tissue but was absent in healthy controls. Importantly, more than 65% of CRP positive cells co-expressed vimentin, confirming them as FLSs. Furthermore, RA synovial cells expressed high levels of CRP receptors CD32 and CD64, which were minimally expressed in controls. Mechanistically, CRP induced inflammatory signaling through activation of the CD32/CD64-p38 and NF-κB pathways. CRP stimulation led to increased phosphorylation of p38 mitogen-activated protein kinase (p38 MAPK) and NF-κB, while blockade of CRP signaling using anti-CD32 or dual anti-CD32/CD64 antibodies significantly reduced this phosphorylation. These results identify the CD32-p38/NF-κB axis as a key signaling pathway mediating CRP-induced FLS activation in RA. In addition, the use of the NF-κB inhibitor pyrrolidine dithiocarbamate (PDTC) effectively suppressed CRP-induced proliferation of RA-FLSs, supporting the therapeutic potential of targeting this pathway. Collectively, these findings suggest that local CRP production by FLSs contributes to synovial inflammation in RA and that inhibition of CRP signaling may offer a novel strategy for disease management [40] (see Figure 1).

### 2.4. Pro-Inflammatory Mechanisms of mCRP in RA

A recent study by Thomé et al. explored the interaction between CRP isoforms, particularly mCRP, and T cells, revealing a complex, context-dependent relationship. While both mCRP and pCRP bound to T cells, only mCRP induced increased proliferation and reduced apoptosis, particularly in activated T cells. However, mCRP did not directly activate T cells, as evidenced by unchanged expression of activation markers CD69 and CD25, and even reduced activation under CD3/CD28 co-stimulation. Notably, the proinflammatory effects of mCRP on T cells only emerged in the presence of monocytes, indicating a monocyte-dependent activation mechanism, likely mediated by mCRP-induced upregulation of CD80 on monocytes, leading to T cell activation via the CD80/CD28 co-stimulatory pathway. This mechanism was confirmed by the use of Belatacept (Bristol-Myers Squibb, East Syracuse, New York, USA), which blocked the pathway and suppressed T cell activation. These findings suggest that mCRP indirectly promotes T cell activation through monocyte interaction, with significant implications for immune responses in settings like allograft rejection and autoimmune diseases [41].

Apoptotic cells, when properly cleared by macrophages, generally induce anti-inflammatory cytokines like TGF-β, while necrotic cells trigger proinflammatory responses. A study by Gershov et al. demonstrated that CRP bound specifically to the membranes of apoptotic and not necrotic cells in a calcium-dependent manner, likely targeting altered membrane lipids such as lysophospholipids. This binding enhanced classical complement pathway activation (notably C1q and C3b deposition) without leading to the formation of the membrane attack complex (MAC), thereby promoting phagocytosis without cell lysis. Evidence indicates that CRP achieves this by recruiting factor H, which inhibits late complement components. Elevated CRP levels also boosted macrophage uptake of apoptotic cells and sustained anti-inflammatory TGF-β expression; however, in the absence of C1q, CRP failed to suppress proinflammatory TNF-α and may therefore even exacerbate inflammation, emphasizing the importance of C1q and CRP working together to ensure non-inflammatory clearance of dying cells and suggesting a mechanism linking deficiencies in early complement components and CRP to autoimmune diseases such as SLE. Their work concluded that inadequate clearance of apoptotic cells, especially in tissues with ongoing inflammation, may result in secondary necrosis, self-antigen presentation, and breakdown of immune tolerance, contributing to autoimmune disease progression [42].

mCRP has also been shown to trigger inflammation in OA both in vitro and in vivo. In human chondrocytes from healthy donors, mCRP promoted a pro-inflammatory environment, with effects comparable to those induced by LPS, a known trigger of inflammation. Similarly, in ATDC5 cells, mCRP significantly increased nitrite production and upregulated several inflammatory and catabolic markers. Also, by using chondrocytes from OA patients and healthy individuals, mCRP exerted persistent, multigenic inflammatory effects across different conditions, highlighting the broad and sustained activity of mCRP in promoting inflammation, even in chondrocytes not previously exposed to a diseased environment [43]. Similarly, the potential contribution of CRP to the development of metabolic OA was investigated using a human CRP-transgenic mouse model fed a high-fat diet. Compared to wild-type mice, human CRP-transgenic males developed more severe OA, with greater cartilage damage and osteophyte formation. Although both groups showed similar levels of systemic inflammation and synovitis, human CRP-transgenic mice had increased activation of monocytes, suggesting enhanced immune cell recruitment as a possible mechanism. The findings support the idea that CRP plays an active role in worsening metabolic OA and could be a target for therapeutic intervention [44].

A report by Rajab et al. introduced a new perspective on interpreting diagnostic CRP levels by highlighting the link between CRP and inflammation-related tissue damage. They highlighted the importance of the potential energy released when the pentamer dissociates, summarizing that mCRP was the true “prototypical acute phase reactant,” characterized by strong, localized, and short-lived proinflammatory effects. During the early stages of the host defense response to tissue injury, any available pCRP is rapidly converted into mCRP. As the production of mCRP declines or the protein is degraded by proteases, the acute inflammatory response transitions into a chronic phase. pCRP begins to accumulate in the bloodstream only after the (rate-limiting) conversion to mCRP slows, typically 6 to 10 h after the initiating event. Therefore, elevated blood levels of pCRP are more indicative of a chronic inflammatory state. If this chronic response is sustained or severe, it could lead to further tissue damage through prolonged neutrophil activity and the release of reactive oxygen species and hydrolytic enzymes into affected tissues [45].

Conformational change in CRP leads to the exposure of neo-epitopes that drive leukocyte activation, oxidative stress through reactive oxygen species (ROS), and leukocyte–endothelial interactions, particularly via lipid raft-mediated signaling pathways [26]. Furthermore, mCRP exhibits unique structural and functional properties that influence neutrophil behavior. Unlike nCRP, mCRP upregulates neutrophil CD11b/CD18 expression and enhances their adhesion to activated endothelial cells, promoting inflammatory responses at vascular injury sites [46]. Since ECs are more responsive to apical (blood-facing) stimulation than basolateral (tissue-facing), exposure to tissue-resident mCRP, often produced in inflamed sites, may not efficiently activate ECs, especially during chronic local inflammation, such as that which occurs in atherosclerosis, but may in fact have protective effects early in plaque development by promoting non-inflammatory clearance of damaged cells and inhibiting foam cell formation. This was explained by virtue of a polarized EC response caused by an uneven distribution of lipid rafts across the cell membrane, which demonstrated apical enrichment. In chronic settings, mCRP may therefore act mainly as a pattern recognition molecule, whereas in acute inflammation, circulating mCRP may amplify responses through endothelial, platelet, and neutrophil activation [47].

Eisenhardt et al. also examined how mCRP and pCRP affected inflammation in immune cells and showed that both forms of CRP could trigger pro-inflammatory responses, but mCRP had much stronger and distinct effects compared to pCRP. mCRP appeared to play a more active role in promoting inflammation, especially in processes linked to atherosclerosis, such as monocyte activation and adhesion. While pCRP has sometimes been associated with anti-inflammatory effects, this study suggested it may also contribute to inflammation under certain conditions. The results support the idea that mCRP and pCRP have separate and important roles in the development of vascular disease [48].

The possible role of CRP in other autoimmune conditions related to RA, such as systemic lupus erythematosus (SLE), has also been investigated. For example, a study by Karlsson et al. investigated EV-bound CRP isoforms and anti-CRP autoantibodies in SLE, revealing that EVs carrying mCRP were elevated in patients with active disease. Unlike pCRP, which showed no correlation with disease activity whether in serum or on EVs, mCRP+ EVs were associated with heightened disease activity, particularly in cases of lupus nephritis (LN), and this contributed to inflammation and the generation of anti-CRP autoantibodies. The study by Karlsson et al. found an inverse relationship between mCRP+ EV abundance and disease duration, especially in patients with active disease and LN, suggesting their relevance in early disease stages. Interestingly, patients with organ damage had lower mCRP+ EV levels, possibly reflecting reduced disease activity over time or redistribution of CRP to other sites such as the endothelium. The study underscored a potential pathogenic role of mCRP+ EVs in SLE through their involvement in inflammation and autoantibody generation [49].

### 2.5. CRP in RA Under Treatment

RA evolves with flares, especially in cases where treatment is insufficient. The RADAR study investigated RA flares occurring outside of clinical settings using weekly dried blood spot (DBS) sampling. The study enrolled 100 RA patients with active disease who were receiving conventional synthetic DMARDs (csDMARDs). At both baseline and 6 weeks, CRP levels were measured via venipuncture and DBS collected using a finger lancet. Of the 100 participants, 30 patients also collected weekly DBS samples at home during the 6-week follow-up period and mailed them in. These patients recorded self-reported flares, which were defined as stiffness and/or pain in one or more joints lasting over 24 h. CRP was analyzed from DBS samples using ELISA, while venipuncture samples were tested using a reference immunoturbidimetric assay. The findings showed that although increases in CRP alone did not reliably predict impending RA flares, the weekly DBS CRP data collected at home provided greater specificity in identifying flares compared to CRP data from hospital visits. However, DBS CRP values above 25 mg/L (five times the upper limit of normal) were found to be imprecise [50]. The first line of treatment in RA are csDMARDs; a systematic review of 23 papers found that CRP > 7.1 mg/L was predictive of poor response to csDMARDs and CRP ≥ 3 mg/dL was predictive of bone erosion and cartilage destruction. Other biomarkers such as rheumatoid factor (RF) and anti-cyclic citrullinated protein (anti-CCP) were predictive of ultrasound-detected bone erosion and increased radiographic damage, respectively; targeting specific biomarkers for the desired therapeutic effect could bring healthcare a step closer to precision medicine [11]. Interestingly, mCRP may also have implications in osteoarthritis (OA). Liang et al. found that mCRP levels correlated with OA severity, suggesting that mCRP could serve as an early diagnostic marker. Since OA shares some early-stage synovial features with RA, identifying mCRP in joints could facilitate earlier intervention, which is especially important because OA is often diagnosed only after irreversible radiologic changes appear [51].

According to Pope et al., various factors, including body fat, hormone levels, diet, and stress, can also influence CRP concentrations. Elevated CRP is associated with joint damage, including bone erosions, emphasizing its relevance in monitoring disease progression. RA is not limited to joint involvement; it is a systemic condition associated with comorbidities such as cardiovascular disease, pulmonary complications, metabolic syndrome (e.g., diabetes), and depression. RA patients have approximately twice the risk of CVD compared to the general population (reviewed in [2]).

## 3. CRP Informs CVD Risk in RA Patients

Atherosclerosis and congestive heart failure (CHF) are among the most common cardiovascular complications observed in patients with RA. The immune system plays a central role in the development of these conditions, as proinflammatory cytokines involved in RA also promote atherogenesis. Several studies reviewed have shown that higher RA disease activity, which is closely linked to elevated levels of pCRP, is associated with poorer cardiovascular outcomes and the presence of unstable atherosclerotic plaques. Inflammation not only drives the formation of these plaques but also contributes to their instability, increasing the risk of rupture and subsequent cardiac events [52].

### 3.1. Key Molecular Mechanisms of RA-Associated CVD

RAGE is a member of the immunoglobulin superfamily of cell surface receptors and has been shown to play a role in the inflammatory processes underlying both RA and CVD; its activation occurs through binding of various proinflammatory ligands, notably the S100 protein family, including S100A8/A9 (calprotectin) and S100A12, both of which are overexpressed in RA and psoriatic arthritis (another autoimmune disease affecting the joints). Chen et al. showed that RAGE was significantly upregulated at sites of severe vascular injury and inflammation and was implicated in the amplification of inflammatory cascades, thereby contributing to the pathogenesis of RA. The study involved 138 patients with RA and 44 healthy controls and assessed the relationship between inflammatory biomarkers and CV risk. The serum levels of S100A8, S100A9, and S100A12 were measured using in-house ELISAs, while soluble RAGE (sRAGE) was quantified with an ELISA-based immunoassay. sRAGE lacks the transmembrane and cytosolic signaling domains; therefore, it has a protective role by acting as a decoy receptor, thereby inhibiting downstream inflammatory signaling. Clinical data included CV event history (after RA diagnosis), smoking, diabetes, family history of early CV disease, BMI, blood pressure, hypercholesterolemia, and hypertension. Laboratory tests covered lipid profiles, glucose, creatinine, CRP, erythrocyte sedimentation rate (ESR), anti-citrullinated peptide antibody (ACPA), RF. S100 proteins were significantly elevated in RA patients and correlated with each other, suggesting co-regulation, whereas sRAGE levels did not differ between groups. However, higher sRAGE levels were associated with a more favorable vascular risk profile, except for HDL, which showed an inverse trend. Overall, their findings suggest that sRAGE and S100 proteins are linked not only to RA-related inflammation and autoantibodies but also to traditional CV risk factors and potential vascular damage [4].

Alterations in HDL particle composition and function may help to explain further connections between inflammation and CV risk in RA. In a study by Chang et al., RA patients treated with Janus kinase inhibitors (JAKi), an immune modulating drug, exhibited a significant increase in serum levels of total HDL particle number, small-sized HDL particles, and HDL-related metabolites, including total lipids, phospholipids, cholesterol, cholesterol esters, and free cholesterol, concurrent with decreased disease activity and reduced CRP levels. The atheroprotective capacity of HDL is not solely dependent on quantity but also on particle size and composition. RA patients, particularly those who are ACPA-positive, have been observed to possess a higher proportion of large or very large HDL particles relative to healthy controls. This shift is thought to result from increased activity of phospholipid transfer protein, which promotes phospholipid accumulation and HDL particle enlargement. Importantly, this study revealed an inverse relationship between CRP levels and the concentration of atheroprotective small and medium-sized HDL particles, suggesting that inflammatory status (or CRP itself) directly influences HDL composition and, by extension, its cardioprotective properties [5].

Chronic inflammation in RA promotes a prothrombotic state and contributes to accelerated atherosclerosis. The degree of systemic inflammation, as reflected by biomarkers such as CRP and ESR, has been independently associated with CV mortality, with baseline CRP or ESR within the first year of RA onset being predictive of future CV death [53]. Arterial stiffness, a recognized marker of vascular dysfunction and an independent predictor of cardiovascular disease, has also been investigated in RA. A twelve-week course of atorvastatin was shown to significantly reduce arterial stiffness (a predictor of CVD and vascular dysfunction), registering a 12% reduction, in 29 long-standing RA patients with moderate disease activity, assessed using pulse-wave analysis, although the treatment did not lead to a reduction in CRP levels [54]. However, Albert et al. investigated whether pravastatin, another statin medication, has anti-inflammatory effects as measured by reductions in CRP, an established marker of cardiovascular risk. The study included over 2800 participants; in the primary prevention group, those receiving 40 mg/day of pravastatin experienced a significant 16.9% median reduction in CRP levels at 24 weeks compared to placebo, with reductions evident as early as 12 weeks. Similar CRP reductions were observed in the secondary prevention group. Importantly, these effects appeared to be independent of changes in low-density lipoprotein cholesterol (LDL-C). The findings suggest that statins like pravastatin may exert beneficial anti-inflammatory effects beyond their cholesterol-lowering action [55].

Autoantibodies can be present is pathologies that are not autoimmune in nature. Wetterö et al. examined the involvement of autoantibodies directed against mCRP in the context of CAD, comparing their levels among 140 individuals, including healthy controls and patients diagnosed with either acute coronary syndrome (ACS) or stable angina pectoris (SA). Using immunoassays to quantify circulating anti-CRP antibodies, the results showed a significant reduction in anti-CRP levels among ACS patients, with the lowest concentrations observed in individuals not receiving statin therapy. A modest positive correlation emerged between anti-CRP levels and body mass index (BMI), whereas no associations were found with traditional risk factors such as smoking status or serum cholesterol levels. The proposed mechanism suggests that during acute plaque destabilization in ACS, anti-CRP antibodies are consumed through enhanced opsonization of mCRP deposited within inflamed atherosclerotic lesions, potentially exacerbating local inflammation and tissue damage. The cohort included 90 patients with angiographically confirmed CAD (40 with ACS and 50 with SA), wherein the latter group largely exhibited clinically stable symptoms for over six months. Comparatively higher levels of anti-CRP antibodies in SA patients and healthy controls further support a potential immunoregulatory role for these antibodies in vascular inflammation, underscoring the importance of CRP–autoantibody interactions in the progression of atherosclerosis and cardiovascular risk [56].

Biomarker studies have highlighted vascular dysfunction in RA, with CRP emerging as a central marker linking systemic inflammation to cardiovascular risk. In a study by Pusztai et al. involving RA and ankylosing spondylitis (AS) patients initiating TNF-α inhibitor therapy, CRP levels were significantly associated with several vascular biomarkers, including B-type natriuretic peptide (BNP), oxidized low-density lipoprotein/β2-glycoprotein I (oxLDL/β2-GPI) complexes, soluble urokinase plasminogen activator receptor (suPAR), and antibodies against 60 kDa heat shock protein (anti-Hsp60). Notably, BNP levels were elevated in seropositive compared to seronegative RA patients and were predicted by both baseline and 12-month CRP levels, suggesting that systemic inflammation prior to treatment may shape long-term cardiovascular outcomes. Similarly, persistent CRP elevation after therapy predicted suPAR levels at 12 months, reflecting ongoing vascular inflammation. Although oxLDL/β2-GPI levels significantly decreased with anti-TNF therapy, BNP, suPAR, and anti-Hsp60 levels remained unchanged, reinforcing the complexity of vascular response in RA. Regression analyses further underscored CRP, along with triglycerides, as significant predictors of BNP and suPAR at both time points. Overall, CRP was tightly linked to inflammatory and vascular changes, underscoring its utility not only in monitoring disease activity but also in predicting cardiovascular biomarker trajectories in pathologies such as RA [57].

### 3.2. Role of Cytokines in RA-CVD

**Figure 2:**
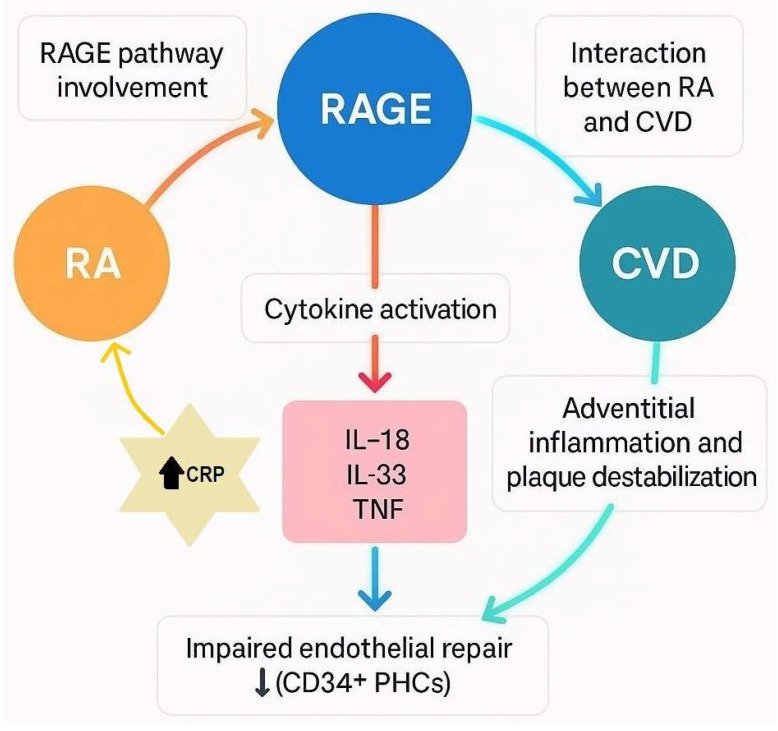
Schematic representation of RAGE, CRP and cytokine involvement in cardiovascular disease related to rheumatoid arthritis. Proinflammatory cytokines such as IL-18, IL-33, and tumor necrosis-factor (TNF) play a role in adventitial inflammation, potentially promoting plaque development and instability, thereby elevating cardiovascular risk in patients with rheumatoid arthritis (RA), particularly those with high C-reactive protein (CRP) levels. The receptor for advanced glycation-products (RAGE) is implicated in the amplification of inflammatory cascades. Notably, RA patients showed markedly increased expression of IL-18 and TNF in the aortic adventitia, along with elevated nuclear IL-33 expression in the endothelial cells of the vasa vasorum.

The presence and role of inflammatory cytokines with potential vascular bioeffects in the aortic adventitia of CVD patients was investigated by Ahmed et al. in order to explore their associations with relevant clinical parameters. The research involved 39 patients undergoing coronary artery bypass graft surgery, 19 with RA and 20 control subjects. Tissue samples were collected from the aortic adventitia, including the epicardial layer, and the internal thoracic artery during surgery. The findings revealed that proinflammatory cytokines such as IL-18, IL-33, and TNF are implicated in the adventitial inflammatory process, potentially contributing to plaque formation and destabilization. Notably, adventitial inflammation involving the vasa vasorum may significantly drive plaque progression and instability, thereby increasing cardiovascular risk in RA patients. The study demonstrated marked alterations in the cytokine microenvironment of the aortic adventitia in CVD patients with RA compared to those without RA. These differences were observed both locally, within the endothelial cells of microvessels in the aortic adventitia, and systemically, in bigger blood vessels such as the internal thoracic artery. Specifically, RA patients exhibited significantly higher expression levels of IL-18 and TNF in the aortic adventitia and increased nuclear IL-33 expression in the endothelial cells of the vasa vasorum. The concentration of soluble ST2 (a protein that plays a role in the immune system and inflammation) showed no correlation with vascular IL-33 expression or other clinical and immunologic markers of RA; however, it was associated with IL-18 expression and CRP levels. Although soluble ST2 may inhibit the activity of circulating IL-33, its elevated systemic levels in RA may indicate heightened inflammation, as suggested by its correlation with CRP levels. Additionally, the association between soluble ST2 and IL-18 expression in the aortic adventitia is noteworthy, given that increased IL-18 expression promotes atherosclerosis, whereas IL-18 deficiency has been shown to reduce it [58] (see Figure 2).

A cross-sectional study analyzing circulating peripheral blood mononuclear cells from RA patients recently identified specific cell subsets that are closely linked to coronary artery calcification. These associations were independent of traditional cardiovascular risk factors and other clinical features of RA. Of note are distinct effector memory CD4 T cell and monocyte subsets, which correlated with coronary artery calcification, indicating a possible intrinsic connection between immune cell profiles characteristic of RA and CVD [59].

Among the molecular signaling factors linking joint inflammation and vascular pathology in RA, OPN has emerged as an important cytokine. OPN not only contributes to joint destruction but also appears to influence arterial stiffness. Elevated circulating OPN levels have been observed in RA patients and are associated with increased aortic pulse wave velocity (PWV), a surrogate marker of arterial stiffness. A study by Bazzichi et al. found that RA patients with OPN levels above the median had significantly higher PWV, indicating a link between joint damage and cardiovascular risk, by looking at 41 RA patients, 28 systemic sclerosis (SSc) patients and 18 healthy controls, excluding subjects with previous CV events. Disease activity and severity were assessed, as well as inflammatory markers such as CRP and ESR, along with quantitative determination of plasmatic OPN using a monoclonal antibody to human OPN. Femoral and carotid sites were used for recording aortic PWV. Their results showed greater CRP and OPN levels in RA patients than the SSc or control groups, as well as significantly increased aortic PWV compared to controls [6].

### 3.3. Endothelial Dysfunction in RA

Endothelial dysfunction, an early sign of atherosclerosis, develops progressively during the course of RA. Södergren et al. investigated whether signs of premature atherosclerosis are present in patients with very early RA. The study spanning 4 years included 79 patients with early RA and 44 healthy controls, focusing on two markers of vascular health: endothelial-dependent flow-mediated dilation (ED-FMD) and carotid intima-media thickness (IMT). The researchers also assessed their relationship with biomarkers of endothelial dysfunction, accounting for systemic inflammation and traditional cardiovascular risk factors. CRP was measured at baseline and every 3 months thereafter; although the levels were raised in the RA group (mean value 13.6 mg/L, with normal values <10 mg/L), there was no relation between CRP (or ESR) and ultrasound findings at baseline. At diagnosis, patients did not show evidence of endothelial dysfunction based on ED-FMD but they did exhibit an increase in IMT over time, suggesting early vascular changes. The findings indicated ongoing endothelial activation in early RA, even in the absence of detectable ED-FMD impairment. Because ED-FMD can fluctuate rapidly, it may reflect acute inflammatory activity when abnormalities are present. Importantly, biomarker differences suggested that endothelial activation in early RA is driven by the disease itself, rather than by acute-phase inflammation. Both IMT and ED-FMD were associated with specific biomarkers of endothelial activation (monocyte chemotactic protein-1 and soluble L-selectin), suggesting that endothelial activation begins very early in RA and may precede the development of measurable endothelial dysfunction and premature atherosclerosis [60].

Crawford et al. revealed a significant increase in microparticles (MPs) of endothelial origin bearing mCRP in patients with peripheral artery disease (PAD), suggesting a role for endothelial-derived MPs in vascular inflammation. Unlike pCRP, which exhibits anti-inflammatory properties, mCRP has been shown to promote inflammation, particularly in models of leukocyte transendothelial migration and maturation. This inflammatory effect is thought to be mediated through the shedding of endothelial MPs. Notably, free mCRP is not detectable in circulation due to its poor aqueous solubility; however, both mCRP and pCRP bound to MPs can be quantified using an image-based cytometric method [61]. mCRP tends to dissociate in lipid environments, particularly in modified lysophospholipids, by overcoming electrostatic interactions [62]. Statin treatment has been shown to reduce mCRP levels in control subjects, highlighting its pro-inflammatory nature. Importantly, the commonly used high-sensitivity CRP (hsCRP) assay did not measure either mCRP or pCRP when bound to MPs. These findings suggest that mCRP may establish a self-sustaining proinflammatory loop through the induction of endothelial MP production, contributing to the chronic vascular inflammation observed in PAD [61].

### 3.4. Vascular Elasticity in RA

One of the earliest detectable changes in the vasculature of RA patients is increased arterial stiffness, even in the absence of CV disease. This phenomenon has been closely linked to persistent systemic inflammation and endothelial dysfunction. In RA, the vascular endothelium becomes dysfunctional due to elevated levels of endothelin-1 and decreased bioavailability of nitric oxide (NO), a vasodilator. Reduced NO levels promote leukocyte adhesion and transmigration, leading to the activation of endothelial cells and upregulation of adhesion molecules, along with increased production of monocyte chemoattractant protein-1 (MCP-1). This cascade facilitates the recruitment of inflammatory cells and cytokines, including IL-2, IL-18, and interferon-γ, which contribute to vascular remodeling, smooth muscle proliferation, and vascular fibrosis, leading to arterial stiffening [63].

Functional vascular changes have been documented through multiple imaging modalities in RA patients. A study involving 75 RA patients and 68 matched controls employed speckle tracking carotid strain (STCS) and ultrasonography to evaluate subclinical vascular dysfunction. The RA group exhibited higher systolic blood pressure, increased arterial stiffness parameters, reduced vessel compliance and strain compared to controls. These changes were present even in the absence of increased carotid intima-media thickness, suggesting that functional abnormalities may precede structural changes [64]. Small artery elasticity measured through pulse-wave analysis was also decreased in 49 longstanding RA patients and found to be inversely related to hsCRP, further emphasizing the role of inflammation in early vascular dysfunction, which was present before IMT thickening in the patients studied [65]. In contrast, in a comparative echocardiographic study of 114 RA patients, 50 individuals with spondyloarthritis, and 50 healthy controls, a significant decline in aortic elasticity was observed in the RA cohort. While arterial stiffness increased with age across all groups, age emerged as the most significant predictor of atherosclerosis. Interestingly, CRP levels did not correlate with aortic stiffness, suggesting that age-related vascular changes may occur independently of systemic inflammation in certain subsets of RA patients [66].

Coronary microvascular dysfunction (CMD) can be measured by myocardial flow reserve (MFR), which is equal to the ratio of stress over rest myocardial flood flow, and it has also been investigated in RA patients without apparent CVD. The Liira (Lipids, Inflammation and Cardiovascular Risk in RA) study conducted from 2016 to 2021 included 66 RA patients over 35 years of age (who had no coronary artery disease or hyperlipidemia and with a mean duration of RA of 7.4 years) with active disease about to initiate anti-TNF-α therapy. Using MFR measured by cardiac perfusion PET scans, the study found no significant change in mean MFR or high-sensitivity cardiac troponin T (hscTnT) after 24 weeks of therapy. However, reductions in hsCRP and interleukin-1β levels were associated with lower hscTnT, suggesting a link between reduced inflammation and improved myocardial stress markers [67].

### 3.5. Atherosclerosis and Endothelial Repair in RA

Giles et al. examined patients with RA and subclinical atherosclerosis and found that both swollen joints count (assessed through clinical examination) and CRP levels were associated with carotid plaque progression. The study included RA patients aged 45–84 years with at least six months since diagnosis and no prior cardiovascular events. Of the 197 participants at baseline, 186 attended the second visit and 158 the third, with carotid ultrasonography performed during visits 1 and 3, and clinical data and biospecimens collected at all visits. Several RA characteristics were linked to site-specific changes in subclinical atherosclerosis, with patients in the early stages of disease showing more rapid progression of maximum common carotid artery intima-media thickness (maxCCA-IMT), a result influenced by baseline cardiovascular risk and statin use. Treatment with TNF-α inhibitors was associated with significantly slower maxCCA-IMT progression compared to those not receiving TNF inhibitors, a benefit not observed with other DMARDs (drugs used in disease control). Notably, higher systemic inflammation as indicated by elevated average CRP and IL-6 levels was predictive of new or progressive plaques, even in patients with few swollen joints (low disease activity), suggesting that longitudinal monitoring of inflammatory markers may improve cardiovascular risk assessment and guide therapy adjustments, even when joint symptoms appear well controlled. Overall, these findings underscore that early RA, systemic and articular inflammation may accelerate subclinical atherosclerosis progression [68]. Diastolic dysfunction is more prevalent in patients with rheumatoid arthritis and is linked to elevated levels of circulating IL-6 [69]. To further support these findings, a three-year cohort study of 487 rheumatoid arthritis patients undergoing serial carotid ultrasonography found that both systemic inflammation, measured by ESR, and traditional cardiovascular risk factors were associated with accelerated progression of atherosclerosis, as indicated by increased IMT [70].

In addition to inflammatory cytokines and lipid-related markers, endothelial repair capacity is impaired in RA. Circulating CD34+ hematopoietic progenitor cells (PHCs), which play a central role in endothelial regeneration, were found to be significantly reduced in RA patients compared to healthy controls in a study by Lo Gullo et al., in which 24 RA patients and 26 matched controls took part. The diminished CD34+ cell count may compromise vascular repair mechanisms and partially explain the increased CV morbidity and mortality observed in RA [7]. Interestingly, systemic inflammation alone, as reflected by elevated hsCRP, may pose a CV risk even in the absence of autoimmune disease. A study by Li et al. found an association between coronary artery tortuosity and elevated hsCRP levels in hypertensive patients, suggesting that systemic inflammation contributes to vascular pathology and the risk of lacunar stroke independently of traditional atherosclerotic changes [71].

### 3.6. CRP Implicated in Metabolic Syndrome

Ridker et al. investigated the relationship between CRP, the metabolic syndrome (defined by the presence of at least 3 of 5 risk factors: upper-body obesity, high triglycerides, low HDL, high blood pressure, and abnormal glucose) and cardiovascular events in 14,719 healthy women over an 8-year period. Metabolic syndrome was present in 24% of participants at baseline, with CRP levels rising progressively with the number of metabolic syndrome components. Cardiovascular risk was elevated in those with high CRP levels (≥3.0 mg/L), regardless of the number of metabolic syndrome traits. Notably, CRP provided additional predictive value for cardiovascular events even among those already diagnosed with metabolic syndrome. The study concluded that measuring CRP enhances risk assessment beyond traditional metabolic syndrome definitions [72]. Another large prospective study investigated the value of CRP in predicting coronary heart disease (CHD). Researchers measured CRP and other inflammatory markers in patients who had coronary events and in controls without such events, using samples taken about 12 years apart to assess stability over time. CRP levels showed moderate long-term stability similar to blood pressure and cholesterol. After adjusting for established risk factors, higher CRP levels were associated with an increased risk of CHD. The study suggests that while CRP has some predictive value, its role in assessing CHD risk should be reconsidered [73].

According to a cohort study that investigated whether hsCRP levels predict the development of diabetes in a Norwegian adult population over a 7-year period, elevated hsCRP was an independent predictor of future diabetes risk. Among 8067 participants without diabetes at baseline, 4% developed diabetes by follow-up. After adjusting for demographic, behavioral, and cardiovascular factors including obesity and hypertension, those in the highest hsCRP tertile had a 73% higher risk of developing diabetes compared to those in the lowest tertile. The association remained significant even after accounting for obesity and hypertension, with no interaction found by sex, BMI, hypertension, or abdominal obesity [74]. Therefore, inflammation could play a role in diabetes development or at least be a predictive factor.

Rethorst et al. investigated the interrelationships among depressive symptoms, systemic inflammation, obesity, and metabolic syndrome (MetS) in a large U.S. adult population sample, employing the PHQ-9 questionnaire to assess depression severity, with a threshold score of ≥10 indicating clinically significant symptoms. Inflammatory status was quantified through serum CRP levels using established clinical cutoffs of ≥3.0 mg/L (a recognized cardiovascular risk marker) and ≥5.0 mg/L (suggested as a predictor of poor antidepressant response). Among the 5579 participants, 606 (11%) met criteria for clinically significant depression, and CRP data were available for 585 of these individuals. Notably, 47% of the depressed subgroup had CRP levels ≥3.0 mg/L, and 29% exceeded 5.0 mg/L, reflecting a substantial burden of low-grade inflammation. CRP levels did not differ significantly across sex, age, or race, but demonstrated strong positive correlations with anthropometric and metabolic markers, including BMI, waist circumference, insulin resistance, and glucose levels. Of the depressed individuals who underwent fasting assessments, nearly half (48.5%) fulfilled diagnostic criteria for MetS. Furthermore, elevated CRP was significantly associated with increased prevalence of key MetS components such as central adiposity, hyperglycemia, and reduced HDL cholesterol. These findings reinforce the concept that inflammation, as indexed by CRP, may serve as a biological bridge linking depression and cardiometabolic risk, with implications for integrated treatment strategies targeting both psychological and metabolic health [75].

The chronic inflammation inherent to RA plays a central role in driving cardiovascular disease through multiple interconnected pathways. Despite therapeutic advances, persistent inflammatory activity, evidenced by elevated CRP, continues to contribute to vascular dysfunction and increased CV risk, underscoring the need for targeted strategies that address both joint and vascular inflammation in RA management.

## 4. Neurocognitive Implications in RA Related to CRP

### 4.1. AD, Dementia, Stroke and CRP

AD is a progressive neurodegenerative disorder that primarily affects the elderly population. Pathological features of AD include the presence of senile plaques (SPs) and neurofibrillary tangles (NFTs) in the brain. Senile plaques are largely composed of aggregated Aβ protein, while NFTs are composed of phosphorylated microtubule-associated tau protein. Mounting evidence points to a substantial role of neuroinflammation in the pathogenesis of AD, with CRP, a key inflammatory biomarker, implicated in this process. CRP has been observed in association with both Aβ and tau lesions in the brains of AD patients, suggesting a potential pathogenic role beyond mere systemic inflammation. CRP is known to activate complement, potentially exacerbating local inflammatory responses within the brain. This has led researchers to consider CRP not just a marker but a possible trigger of AD-like dementia. Supporting this, a study by Bi et al. demonstrated that intracerebroventricular injection of CRP in rats induced significant impairments in learning and memory, reinforcing the hypothesis that CRP may contribute directly to neurodegenerative processes [8].

Systemic inflammation arising from conditions such as RA, infections, and sepsis exerts significant effects on multiple tissues, with the blood–brain barrier (BBB) functioning as a key interface mediating the relationship between RA and AD. Immune hyperactivation in RA leads to elevated concentrations of pro-inflammatory signaling molecules that compromise BBB integrity and increase its permeability. Empirical evidence demonstrates altered BBB permeability in RA patients, a phenomenon also implicated in neurodegenerative diseases including AD. Inflammatory cytokines, such as IL-6, IL-12, CRP, pentraxin 3, endothelin-1, resistin, and RAGEs, readily cross the BBB via several mechanisms: passage through periventricular organs lacking a complete barrier, activation of the vagus nerve, and direct binding to endothelial cells, causing tight junction disruption and cytokine penetration into cerebral tissue. These cytokines play pivotal roles in modulating BBB tightness by downregulating occludin and other tight junction proteins, thereby diminishing barrier integrity. Both RA and AD share pathological amyloid involvement: AD is characterized by accumulation of Aβ plaques leading to neuronal excitotoxicity, synaptic protein loss, and cholinergic dysfunction, whereas in RA, cytokine-driven amyloid deposition contributes to bone and joint matrix degradation. Epidemiologically, AD prevalence is elevated in RA populations compared to healthy controls, indicating a complex interplay among the nervous, skeletal, and immune systems, as well as aging processes. However, despite overlapping inflammatory markers and pathways, the causative relationship between RA and AD remains ambiguous. It is plausible that distinct inflammatory cascades underlie each condition, with shared biomarkers reflecting convergent immune activation rather than direct pathogenesis. This necessitates further mechanistic studies to elucidate immune signaling pathways in RA-associated AD and in comorbid cases [76].

A study by Strang et al. used specific antibodies to analyze Aβ plaque formation and its interaction with different proteins. Artificial Aβ plaques were prepared and examined through various laboratory techniques, including Western blot and immunohistology. Human brain tissues from AD patients and controls were analyzed for the presence and localization of plaques and related proteins. Quantification and co-localization assays were performed to study differences between AD and non-AD samples. Artificial Aβ plaques formed in vitro dissociated pCRP into mCRP, a process not observed with non-aggregated Aβ peptides. Immunohistological staining confirmed strong mCRP presence within these plaques, while pCRP was barely detectable, indicating that plaque formation promotes CRP dissociation. The artificial Aβ42 plaques mimic AD brain plaques by forming beta-sheet structures, as shown by Thioflavin T fluorescence. Importantly, brain tissue sections from AD patients revealed mCRP co-localized with β-amyloid plaques, whereas pCRP was absent; non-AD controls showed no such staining. Different antigen retrieval methods verified that pCRP dissociation was not an artifact of staining. Furthermore, complement protein C1q was found to co-localize with mCRP in AD brain sections, suggesting a potential interaction between mCRP, amyloid plaques, and the complement system in AD pathology. This evidence supports the notion that mCRP, generated from pCRP upon interaction with Aβ plaques, plays a significant role in AD [77].

While the shared inflammatory mechanisms between RA and AD are scientifically compelling, the current body of evidence does not support a direct causal relationship between the two conditions. For instance, the presence of mCRP within Aβ plaques and its co-localization with complement proteins such as C1q in AD brain tissue suggest a potential role in local immune activation and neuroinflammatory processes [77]. However, these findings are primarily associative and do not establish mCRP as a pathogenic driver of AD. Similarly, elevated systemic inflammation in RA may contribute to blood–brain barrier dysfunction and heightened neuroinflammatory states, but it remains unclear whether this directly initiates or accelerates AD pathology. These observations should therefore be framed as hypothesis-generating rather than conclusive. Additional studies are needed to determine whether these shared inflammatory features represent overlapping but independent pathways or reflect a true pathogenic bridge between RA and AD.

Kanimozhi et al. investigated the link between periodontal disease and chronic autoimmune, inflammatory, and neurodegenerative disorders. The researchers divided 200 patients into 4 groups of 50, corresponding to RA, AD, Parkinson’s disease, and moderate to severe periodontal disease. They assessed systemic inflammatory markers such as CRP and IL-6. Among all groups, individuals with periodontal disease (an inflammatory condition caused by microbial buildup in the oral cavity and measured through clinical attachment loss, bleeding on probing, and probing pocket depth) showed the highest levels of systemic inflammation. These results highlight the growing recognition that chronic inflammation such as that arising from periodontitis may contribute to or exacerbate systemic autoimmune and neurodegenerative conditions via inflammatory pathways involving CRP and cytokines such a IL-6 [9].

Cardiovascular health has also been implicated in AD pathogenesis. A study by Stamatelopoulos et al. involving 1464 patients across two clinical centers examined markers such as amyloid beta 1-40 (Abeta40), hsCRP, and high-sensitivity troponin T (hsTnT), alongside coronary angiography. Over a five-year follow-up, Abeta40 was independently associated with major adverse cardiac events (MACE), cardiovascular death, and progressive aortic and arterial stiffness. Furthermore, increasing Abeta40 levels correlated with rising hsCRP, hsTnT, reduced glomerular filtration, and decreased left ventricular ejection fraction, strengthening the proposed link between systemic vascular inflammation, amyloid pathology, and cognitive decline [78]. Further evidence linking CRP to neurodegeneration comes from postmortem studies in dementia and AD patients who experienced stroke. Monomeric CRP was identified within Aβ senile plaques, particularly in infarcted and peri-infarcted regions. Notably, mCRP staining was strongest in penumbral regions and ischemic microvessels, areas often associated with active or ongoing injury. Conversely, neurodegenerative regions without overt infarction showed minimal mCRP presence, highlighting a possible spatial relationship between vascular injury, inflammation, and plaque formation [79].

**Figure 3:**
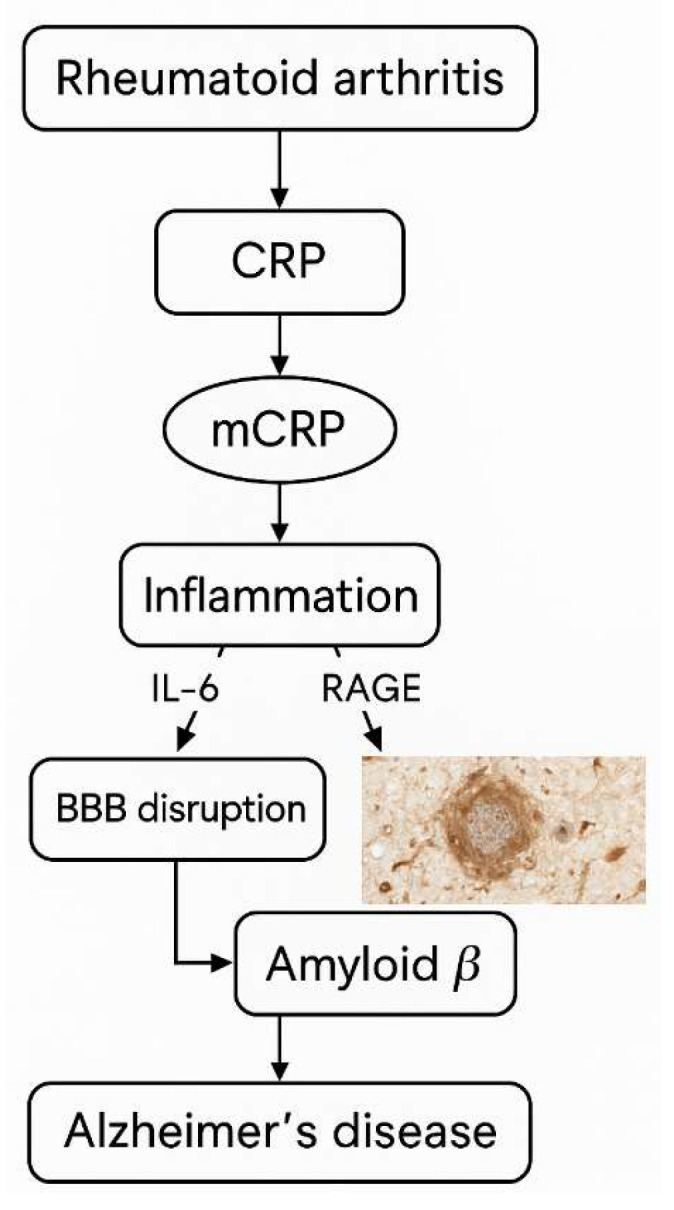
Schematic representation of monomeric CRP’s role in angiogenesis post-stroke. This figure illustrates the proposed mechanistic link between rheumatoid arthritis (RA) and Alzheimer’s disease (AD), emphasizing the central role of C-reactive protein (CRP) and its monomeric form (mCRP) in driving systemic and neuroinflammation. In RA, chronic immune activation leads to elevated systemic levels of CRP and interleukin-6 (IL-6), which contribute to vascular inflammation and endothelial dysfunction. These inflammatory mediators, along with receptor for advanced glycation end-products (RAGE), facilitate blood–brain barrier (BBB) disruption, allowing peripheral inflammatory molecules to access the central nervous system. As BBB integrity diminishes, CRP crosses into the brain and interacts with amyloid-beta (Aβ) plaques. Experimental data support the idea that Aβ promotes the dissociation of pentameric CRP into pro-inflammatory mCRP, which co-localizes with plaques and complement component C1q, thereby exacerbating neuroinflammatory processes and neuronal injury. The accumulation of Aβ and mCRP in cerebral tissue promotes further amyloidogenesis, complement activation, and neuronal damage, hallmarks of AD pathophysiology. The diagram also incorporates findings from stroke research, where ischemic injury and hypoxia lead to local mCRP production in brain microvessels and neurons. Post-stroke angiogenic responses and increased mCRP expression may create a vulnerable neurovascular environment, predisposing individuals to progressive neurodegeneration. Collectively, the figure highlights the overlapping pathways of inflammation, vascular dysfunction, and amyloid pathology linking RA, systemic CRP/mCRP activity, BBB disruption, and AD progression.

In another study, brain tissue was analyzed from deceased patients to better understand the changes that occur in the brain following a stroke. Tissue samples were collected shortly after death and analyzed to examine cellular behavior, protein expression, and the formation of new blood vessels under both normal and stress conditions. A key focus of the study was the role of different forms of CRP in brain recovery and inflammation. The findings revealed that CRP, especially its monomeric form, plays a significant role in post-stroke brain changes, particularly by promoting or regulating angiogenesis. Immunohistochemical analysis showed that mCRP was predominantly expressed in the microvessels of infarcted and peri-infarcted brain tissue in ischemic stroke patients. It co-localized with markers of active angiogenic endothelium, whereas the native pentameric form was only minimally present. Additionally, mCRP was found within the nuclei and cytoplasm of stroke-affected neurons, particularly in dying cells. Western blot analysis further confirmed increased mCRP expression in stroke-affected regions. Complementary in vitro experiments involving oxygen–glucose deprivation demonstrated that hypoxic conditions led to increased intracellular mCRP and de novo CRP synthesis in human neurons and brain endothelial cells. These results suggest that mCRP is involved in the cellular response to ischemia and may contribute to toxic inflammatory and aberrant angiogenic effects after stroke that could create increased susceptibility to development of AD [80] (see Figure 3).

### 4.2. Cognitive Dysfunction and CRP

Rheumatoid arthritis has also been associated with cognitive dysfunction. In a cohort of 60 female RA patients (20 on methotrexate and 40 on intravenous biologics), alongside 39 healthy controls, significant cognitive impairment was documented using a number of neuropsychological tests. Among the RA patients, brain MRI revealed pathological cerebral alterations including vascular lesions and cerebral atrophy. These abnormalities were more prevalent among patients than controls and were notably associated with higher disease activity and lower educational status. A strong correlation emerged between cerebral vascular pathology, carotid atherosclerosis, reduced circulatory reserve capacity, and cognitive decline in the RA population [81]. To further support this, a large UK study examined cognitive function in 661 individuals with RA and found a high prevalence of cognitive impairment among them, suggesting that chronic systemic inflammation plays a significant role in this impairment. They speculated that RA disease activity (objectified through DAS28-CRP) could emerge as a potentially modifiable risk factor that could be targeted to help slow or prevent cognitive deterioration [82].

A large Japanese cohort study explored the relationship between serum hsCRP levels and brain volume in 8614 adults aged 65 years and older, utilizing MRI imaging to quantify neuroanatomical changes. Serum hsCRP was measured via latex agglutination turbidimetry and stratified into four clinically relevant categories: <1.0, 1.0–1.9, 2.0–2.9, and ≥3.0 mg/L. Dementia diagnoses were established through expert clinical evaluation. Elevated hsCRP levels were associated with unfavorable baseline characteristics, including older age, lower educational attainment, smoking, higher BMI, and increased rates of hypertension, diabetes, ischemic heart disease, chronic kidney disease, stroke, and ECG abnormalities. The age. and sex-adjusted prevalence of all-cause dementia, Alzheimer’s disease (AD), and non-AD dementias increased progressively across hsCRP categories. Multivariable logistic regression revealed that participants with hsCRP ≥2.0 mg/L had significantly higher odds of all-cause dementia and AD, whereas associations with non-AD dementia were not statistically significant. Moreover, higher hsCRP levels were linked to reduced total brain volume relative to intracranial volume, with prominent atrophy observed in the temporal cortex. These associations remained robust after excluding individuals with dementia and applying multiple comparison corrections, indicating a potential inflammatory pathway contributing to neurodegeneration and Alzheimer’s risk in older adults [83].

Natale et al. examined the interplay between systemic inflammation, cognitive impairment, and sociodemographic factors in a cohort of 4601 older adults, stratifying participants into four diagnostic categories: cognitively unimpaired, probable AD, probable major stroke, and probable mixed AD, based on longitudinal episodic memory trajectories analyzed via a pattern recognition algorithm. Inflammatory status was assessed using CRP measured from dried blood spot assays, with CRP values exceeding 30 mg/dL excluded in case of probable acute infection. The study incorporated a broad range of covariates, including race/ethnicity, age, sex, educational status, cardiovascular comorbidities, obesity (BMI ≥30), smoking status, alcohol use, and depressive symptoms, the latter quantified using the CES-D-8 scale, with scores ≥3 indicating possible depression. Individuals with probable AD, mixed AD, or major stroke exhibited significantly elevated CRP levels relative to cognitively unimpaired counterparts, with the greatest elevations observed in the mixed AD group. Elevated CRP was positively associated with known inflammatory risk factors such as diabetes, hypertension, smoking, obesity, and Black race. Conversely, protective associations were identified among non-depressed individuals reporting higher educational status and alcohol consumption. Within the depressed cohort, hypertension and obesity remained independently associated with higher CRP, whereas female sex and alcohol intake were linked to lower levels. Interestingly, advancing age correlated with elevated CRP only among non-depressed participants, suggesting a complex and heterogeneous relationship between inflammation, cognitive decline, demographic variables, and affective symptoms in aging populations [84].

Another large retrospective cohort study investigated the association between autoimmune rheumatic diseases (ARDs) and the subsequent development of dementia using Taiwan’s comprehensive National Health Insurance Research Database (NHIRD), which covers nearly 99% of the population. A total of 34,660 patients diagnosed with ARDs between 2001 and 2012 were identified from the catastrophic illness registry, where diagnoses were confirmed by rheumatologist specialists to ensure accuracy. These patients were matched by age and sex with 138,640 individuals without ARDs to form a comparison cohort, and individuals with a prior dementia diagnosis or aged below 45 years were excluded. Each participant was followed from their index date until a confirmed diagnosis of dementia, withdrawal, death, or the end of 2012. Dementia outcomes included AD, vascular dementia, and other unspecified forms. Baseline comorbidities such as diabetes, hypertension, cardiovascular disease, stroke, psychiatric disorders, and traumatic brain injury were accounted for in multivariable Cox proportional hazards models. The findings revealed that dementia incidence was higher in the ARD group (63.08 per 10,000 person-years) compared to the non-ARD group (48.43 per 10,000 person-years), indicating a 23% increased risk. Subgroup analysis showed that patients with secondary Sjögren’s syndrome exhibited the highest risk, followed by primary Sjögren’s syndrome, while RA and systemic lupus erythematosus had more modest associations. Kaplan–Meier survival curves further illustrated a significantly higher cumulative incidence of dementia among ARD patients compared to controls (log-rank test, *p* < 0.001). Taken together, the results suggest a robust and independent link between ARDs and increased dementia risk, suggesting the need for proactive cognitive monitoring and potentially preventive strategies in this vulnerable population [85].

A Swedish longitudinal population-based study investigated the relationship between midlife joint disorders, specifically RA and other arthritic conditions, and cognitive outcomes in later life. Drawing on data from 1449 individuals initially assessed between 1972 and 1987, with follow-up conducted 21 years later, the study employed self-reported measures of joint disease and standardized cognitive assessments. Adjusted ordinal logistic regression revealed that the presence of joint disorders in midlife was significantly associated with poorer cognitive status in later years, with a notably stronger association observed for RA alone. This association persisted when AD was specifically considered as the outcome. These findings implicate chronic inflammation, particularly the persistent systemic inflammation characteristic of RA, as a potential contributor to neurodegenerative processes, further supporting the hypothesis that inflammatory pathways may play a mechanistic role in cognitive decline, and reinforce the relevance of targeting inflammation in efforts to mitigate dementia and AD risk [86].

Several large-scale longitudinal studies and biobanks have investigated the relationship between systemic inflammation and neurocognitive outcomes in RA, though few have specifically assessed CRP isoforms (mCRP and pCRP). The UK Biobank, which includes over 500,000 participants with extensive cognitive, imaging, and inflammatory marker data, has shown that hsCRP is associated with increased risk of cognitive decline and dementia in RA patients, though it does not differentiate CRP isoforms [87,88]. Similarly, the Brigham and Women’s Rheumatoid Arthritis Sequential Study (BRASS), an RA-specific cohort, reported associations between higher systemic inflammation (including total CRP) and subjective cognitive complaints. The Mayo Clinic Biobank and Rochester Epidemiology Project also offer longitudinal RA data with cognitive outcomes, though only total CRP has been measured to date. While the Alzheimer’s Disease Neuroimaging Initiative (ADNI) provides robust imaging and cognitive data, RA patients are underrepresented, and CRP isoform data are lacking [89]. Experimental studies, however, have increasingly implicated mCRP in neuroinflammatory processes, showing its colocalization with amyloid plaques and its capacity to disrupt the blood–brain barrier and activate microglia [79,80]. These findings suggest that mCRP could serve as a mechanistic link between chronic RA-related inflammation and neurodegeneration. Although no longitudinal cohort has yet measured CRP isoforms in this context, stored biospecimens in biobanks such as UK Biobank or BRASS could potentially be reanalyzed using isoform-specific assays to clarify these relationships.

### 4.3. Depression and CRP

Depression has been linked to elevated CRP levels. A study investigated the relationship between serum CRP levels and the development of depressive symptoms following a stroke. A total of 572 patients who experienced an ischemic stroke or transient ischemic attack were enrolled, and their serum CRP levels were measured within 48 h of stroke onset. Depressive symptoms were evaluated in 405 patients on day 8 and in 306 patients 3 months after stroke. The findings showed that patients with more severe depressive symptoms at both time points had higher CRP levels. In univariate analysis, a CRP level greater than 9.2 mg/L was linked to depressive symptoms on day 8, while a CRP level above 4.3 mg/L was associated with depressive symptoms at 3 months. Multivariate analysis revealed that elevated CRP levels were significantly associated with depressive symptoms on day 8 but not at the 3-month mark. Therefore, increased CRP levels have been correlated with more pronounced depressive symptoms shortly after stroke, but their impact appeared to diminish over time [90].

Kojima et al. investigated the interrelationships among depressive symptoms, systemic inflammation, and pain perception in a cohort of 218 individuals diagnosed with RA, employing the Japanese version of the Beck Depression Inventory-II (BDI-II) to assess depression severity and hsCRP assays to quantify inflammation. Comprehensive clinical assessments, including tender and swollen joint counts, were performed during a single visit to minimize temporal variability. The analyses revealed a moderate, positive correlation between depressive symptoms and CRP levels, with each independently and significantly associated with heightened pain intensity, even after adjusting for confounding variables. Notably, the probability of experiencing severe pain increased linearly with both rising BDI-II scores and hsCRP concentrations. Severe pain was disproportionately prevalent among older patients, individuals with lower educational status, greater functional impairment, and a higher count of tender joints. Additionally, elevated scores on the visual analog scale (VAS), CRP, and BDI-II were all significantly and positively correlated with pain severity. These results emphasize that pain in RA is multifactorial in nature, shaped by both inflammatory and psychological pathways, and suggest that optimal pain control may necessitate a dual-focus approach that targets both biological inflammation and comorbid depressive symptoms [91].

To expand beyond just rheumatological autoimmune conditions, a recent review comparing depressive symptoms in RA and multiple sclerosis (MS) identified overlapping patterns suggestive of a shared biological basis, proposing that depression in both conditions may represent a downstream manifestation of common neurohumoral and neuroimmunological dysregulation. Despite the incomplete understanding of the precise pathophysiological mechanisms, converging evidence from biochemical analyses, neuroimaging, and psychosocial studies points toward an inflammatory component underpinning mood disturbance in these autoimmune diseases. In RA, elevated CRP levels have been consistently associated with greater depressive symptom severity, while in MS, increased peripheral inflammation, particularly heightened levels of IL-6 and CRP, appears to differentiate individuals with comorbid depression from those without. These findings suggest that depression in RA and MS is not merely a reactive phenomenon to chronic illness burden but may reflect an intrinsic aspect of disease activity and immune-mediated central nervous system involvement. Such insights suggest a potential utility of anti-inflammatory strategies in mitigating depressive symptoms and call for a more nuanced, biologically informed approach to mental health care in autoimmune disease populations [92].

Chamberlain et al. examined the relationship between CRP and clinical features of major depressive disorder (MDD) by comparing treatment-resistant (*n* = 102), treatment-responsive (*n* = 48), untreated patients (*n* = 48), and healthy controls (*n* = 54). The findings showed that CRP levels were significantly elevated in treatment-resistant patients even after adjusting for BMI, and higher CRP was associated with specific symptoms such as vegetative depressive symptoms, anxiety, higher BMI, and childhood adversity. These results suggest that a subgroup of MDD patients with elevated inflammation and distinct clinical profiles might benefit from targeted anti-inflammatory treatments, highlighting the potential for more personalized approaches in managing depression [93].

Accumulating evidence points towards CRP, especially its monomeric form, as playing an active role in neurodegenerative diseases like AD and dementia. Beyond being a marker of inflammation, CRP is involved in amyloid plaque formation, vascular injury, and cognitive decline. Elevated CRP levels are linked to conditions such as strokes, periodontitis, and depression, with an increased risk of brain atrophy and dementia. These findings suggest that CRP may be a major contributor to the initiation and maintenance of neuroinflammatory processes and represents a potential target for therapeutic intervention.

## 5. Conclusions

CRP and particularly mCRP play a pivotal and conformation-dependent role in the pathogenesis and progression of RA. The dissociation of nCRP into mCRP and the intermediate pCRP* not only activates immune and inflammatory pathways but also contributes directly to joint damage by promoting osteoclastogenesis and FLS activation. mCRP engages with key receptors such as CD32 and CD64 on FLSs, triggering the p38 MAPK and NF-κB pathways, leading to pro-inflammatory gene expression and tissue destruction. CRP’s interaction with EVs and immune complexes boosts or strengthens inflammatory signaling even more. While nCRP may exert protective effects in early or pre-clinical RA stages, mCRP appears to drive disease activity and bone erosion in established RA. The presence of CRP and mCRP in synovial tissue and their influence on both local and systemic inflammation underscore their dual role as both biomarkers and potential therapeutic targets. Understanding the structural dynamics of CRP offers valuable insights into RA pathophysiology and opens avenues for precision medicine approaches aimed at modulating its conformation-specific effects.

The biological interaction and overlap between chronic inflammation, immune dysregulation, and vascular pathology are central to understanding the heightened cardiovascular risk in patients with RA. The studies reviewed highlight a multifactorial process involving inflammatory agents such as CRP, S100 proteins, IL-18, IL-33, TNF, and osteopontin, alongside impaired HDL function, endothelial dysfunction, and altered immune cell profiles. Biomarkers like sRAGE, anti-CRP antibodies, and circulating progenitor cells offer insights into both disease mechanisms and potential therapeutic targets. Notably, systemic inflammation not only accelerates atherosclerosis and arterial stiffness but also undermines vascular repair mechanisms and contributes to metabolic derangements. These findings underscore the importance of early cardiovascular risk assessment in RA, extending beyond traditional risk factors to include inflammation-specific markers and vascular imaging. Targeted anti-inflammatory therapies may confer cardiovascular benefits by modulating these inflammatory pathways. Future research should focus on integrating inflammatory biomarkers into clinical risk stratification models and exploring personalized therapeutic strategies aimed at mitigating CVD risk in inflammatory rheumatic diseases.

Monomeric CRP arises as an active contributor to neurodegenerative processes rather than merely a bystander marker of systemic inflammation. CRP appears to play a multifaceted role in the pathogenesis of AD, dementia, and cognitive dysfunction by promoting amyloid plaque formation, disrupting the blood–brain barrier and amplifying neuroinflammatory and vascular injury responses. Its presence in amyloid-filled brain regions, association with cerebral atrophy, and correlation with cognitive impairment across various inflammatory conditions, including RA, stroke, and periodontitis, reinforces the hypothesis that systemic inflammation, mediated in part by CRP, may accelerate or trigger neurodegeneration. Moreover, the link between elevated CRP and depressive symptoms further supports a neuroimmune interface influencing both mood and cognition. Taken together, the evidence described above suggests that CRP is not only a biomarker but a potential pathogenic agent in neurodegenerative and neuropsychiatric diseases, highlighting the importance of targeting inflammation as a therapeutic strategy to preserve cognitive health and mitigate disease progression.

## 6. Future Perspectives

Future therapies targeting mCRP are being actively explored as innovative interventions for chronic inflammatory diseases such as RA, atherosclerosis, and dementia. The dissociation of pCRP into mCRP plays a crucial role in triggering inflammatory processes associated with major diseases, making its inhibition a key target for chemotherapy. Compounds that have been developed to directly inhibit this dissociation by binding to CRP are phospholipase A2 (PLA2) inhibitors. Enzymes in the PLA2 family include secretory (sPLA2), cytosolic (cPLA2), and lipoprotein-associated (LpPLA2); they play a dual role in inflammation by producing lysophospholipids and arachidonic acid, both linked to CRP activation. Inhibitors for each subtype have been developed, such as darapladib for LpPLA2 and varespladib for sPLA2, but most have failed to demonstrate clinical efficacy in late-stage trials. Despite this, research has identified several well-defined potential targets for preventing CRP dissociation, and recent discoveries of synthetic small molecules that bind to CRP suggest that the development of drugs to counteract CRP-driven inflammation will remain a vibrant and important field of study [94].

In addition, a small-molecule inhibitor, 1,6-bis (phosphocholine)-hexane, was developed to specifically block CRP function by binding and crosslinking its active sites. In a rat model of heart attack, this inhibitor prevented CRP-induced increases in infarct size and cardiac dysfunction, although concerns about immune activation and species differences have limited its clinical potential, and development has been discontinued. Despite this, the finding suggests that targeting CRP could be a promising treatment for reducing tissue damage in heart attacks, strokes, and other inflammatory or tissue-damaging conditions [95].

The most promising selective CRP inhibitor was investigated in a study that targeted the phosphocholine (PC)/phosphoethanolamine (PE) head group interactions on the B-face of the CRP pentamer, which are essential for anchoring CRP to damaged cell membranes and initiating its proinflammatory conformational change to pCRP* and mCRP. The designed tool compound, C10M, mimics the anchoring interactions of PC by coordinating with calcium cations through a phosphonate group (which replaces the native phosphate to avoid nuclease degradation) and forming hydrogen bonds with Glu81, Asn61, and Gln150, while its n-butyl substitutions enhance hydrophobic interactions. In vitro, C10M inhibited pCRP*/mCRP-mediated monocyte adhesion, cytokine expression (TNF, IL-6, IL-1β), platelet-leukocyte aggregation, endothelial ICAM-1 and VCAM-1 upregulation, and neutrophil/monocyte activation. Importantly, C10M prevented pCRP*/mCRP-induced NETosis and ROS generation. In vivo, C10M reduced human CRP deposition, inflammation, and tissue injury in a rat model of renal ischemia-reperfusion injury and improved renal function. In a rat hindlimb transplant model, C10M abrogated pCRP-induced graft rejection, leukocyte infiltration, and CRP tissue deposition. Lastly, C10M reduced pCRP-enhanced phagocytosis of PC-expressing *S. pneumoniae* serotype 27 but had no effect on *E. coli* or zymosan phagocytosis, confirming its specificity for PC-binding CRP interactions. These results establish C10M as a monovalent, selective CRP inhibitor with potent anti-inflammatory and immunomodulatory effects in vitro and in vivo [96].

Despite these new findings, while targeting mCRP holds promise as a therapeutic strategy due to its pro-inflammatory and tissue-damaging roles in various diseases, translating these findings into clinical treatments remains challenging. The development of specific inhibitors that can selectively target mCRP without disrupting the beneficial functions of native pentameric CRP is still in early stages. Furthermore, variability in mCRP expression across different tissues and disease states complicates its use as a universal therapeutic target. More research is needed to determine optimal delivery methods, specificity, and long-term safety of potential mCRP-targeted therapies, even though these novel approaches represent a paradigm shift from general immunosuppression to precision modulation of pathological inflammation, with the potential to significantly improve outcomes in the chronic, debilitating conditions discussed within this review.

## Abbreviations

The following abbreviations are used in this manuscript:

ACPA	Anti-Citrullinated Peptide Antibody
ACS	Acute Coronary Syndrome
AD	Alzheimer’s Disease
ADNI	Alzheimer’s Disease Neuroimaging Initiative
Aβ	Beta-Amyloid (Amyloid-beta)
Aβ42	Amyloid-beta 1–42
Abeta40	Amyloid-beta 1–40
Anti-CCP	Anti-Cyclic Citrullinated Peptide
Anti-Hsp60	Antibodies Against 60 kDa Heat Shock Protein
BDI-II	Beck Depression Inventory-II
BMI	Body Mass Index
BNP	B-Type Natriuretic Peptide
BRASS	Brigham and Women’s Rheumatoid Arthritis Sequential Study
C	Complement System
CAD	Coronary Artery Disease
CD	Cluster of Differentiation
CHD	Coronary Heart Disease
CIA	Collagen-Induced Arthritis
CMD	Coronary Microvascular Dysfunction
CRP	C-Reactive Protein
csDMARDs	Conventional Synthetic Disease-Modifying Antirheumatic Drugs
CV	Cardiovascular
CVD	Cardiovascular Disease
DAS28-CRP	Disease Activity Score 28 (or in 28 joints)-CRP
DBS	Dried Blood Spot
DMARDs	Disease-Modifying Anti-Rheumatic Drugs
EC	Endothelial Cell
ECG	Electrocardiogram
ELISA	Enzyme-Linked Immunosorbent Assay
ESR	Erythrocyte Sedimentation Rate
EV/EVs	Extracellular Vesicles
Fcγ receptors	Fc gamma receptors
FLS	Fibroblast-like Synoviocytes
HDL	High-Density Lipoprotein
hscTnT	High-Sensitivity Cardiac Troponin T
hsCRP	High-Sensitivity C-Reactive Protein
IFN-γ	Interferon-gamma
IL-1β	Interleukin-1 beta
IL-2	Interleukin-2
IL-6	Interleukin-6
IL-12	Interleukin-12
IL-18	Interleukin-18
IL-33	Interleukin-33
IMT	Intima-Media Thickness
JAKi	Janus Kinase Inhibitor
LDL-C	Low-Density Lipoprotein Cholesterol
LPS	Lipopolysaccharide
LpPLA2	Lipoprotein-Associated Phospholipase A2
MAPK	Mitogen-Activated Protein Kinase
MAC	Membrane Attack Complex
MACE	Major Adverse Cardiac Events
MCP-1	Monocyte Chemoattractant Protein-1
MDD	Major Depressive Disorder
MetS	Metabolic Syndrome
MFR	Myocardial Flow Reserve
MHC-II	Major Histocompatibility Complex Class II
MRI	Magnetic Resonance Imaging
MPs	Microparticles
mCRP	Monomeric C-Reactive Protein
MVs	Microvesicles
nCRP	Native (Pentameric) C-Reactive Protein
NK	Natural Killer Cells
NF-κB	Nuclear Factor Kappa-light-chain-enhancer of Activated B cells
NFTs	Neurofibrillary Tangles
NHIRD	National Health Insurance Research Database (Taiwan)
OA	Osteoarthritis
OPN	Osteopontin
PAD	Peripheral Artery Disease
PC	Phosphocholine
PE	Phosphoethanolamine
PET	Positron Emission Tomography
PHCs	Progenitor Hematopoietic Cells (CD34+ cells)
PHQ-9	Patient Health Questionnaire-9
pCRP/pCRP*	Pentameric C-Reactive Protein/Pentameric Symmetrical CRP (with neoepitope exposed)/Conformational Intermediate of CRP
PWV	Pulse Wave Velocity
RA	Rheumatoid Arthritis
RAGE/RAGEs	Receptor for Advanced Glycation End Products
RF	Rheumatoid Factor
ROS	Reactive Oxygen Species
sPLA2	Secretory Phospholipase A2
sRAGE	Soluble Receptor for Advanced Glycation End Products
SA	Stable Angina
S100A8/A9	Calprotectin
SLE	Systemic Lupus Erythematosus
SSc	Systemic Sclerosis
SPs	Senile Plaques
ST2	Suppression of Tumorigenicity 2 (soluble form often denoted as sST2)
STCS	Speckle Tracking Carotid Strain
suPAR	Soluble Urokinase Plasminogen Activator Receptor
TNF	Tumor Necrosis Factor
TNF-α	Tumor Necrosis Factor Alpha
TGF-β	Transforming Growth Factor Beta
VAS	Visual Analog Scale
VEGF	Vascular Endothelial Growth Factor
